# Outcome of staged excision with pathologic margin control in high-risk basal cell carcinoma of the head region^☆^^☆☆^

**DOI:** 10.1016/j.abd.2020.02.009

**Published:** 2020-07-12

**Authors:** Reza Kavoussi, Hossein Kavoussi, Ali Ebrahimi, Nader Salari, Seyed Hamid Madani

**Affiliations:** a Department of Dermatology, Kermanshah University of Medical Sciences, Kermanshah, Iran; b School of Health, Kermanshah University of Medical Sciences, Kermanshah, Iran; c Department of Pathology, Kermanshah University of Medical Sciences, Kermanshah, Iran

**Keywords:** Mohs surgery, Pathology, Skin neoplasms

## Abstract

**Background:**

High-risk basal cell carcinoma involves a significant rate of basal cell carcinoma that requires Mohs micrographic surgery for definitive treatment. Staged excision with pathologic margin control is a simple, accessible, and curative procedure suggested for the treatment of high-risk basal cell carcinoma.

**Objective:**

To evaluate the results of staged excision of high-risk basal cell carcinoma in the head region.

**Methods:**

This interventional study was performed on patients with high-risk basal cell carcinoma, who underwent staged excision until the margins were free of tumor.

**Results:**

A total of 122 patients (47 females and 75 males) with mean age of 57.66 ± 9.13 years were recruited in this study. Nasal and nodular types were the most common of both clinical and pathologic forms, respectively. Further, 89.3 % of cases were cured by staged excision after four years of follow-up. There was a significant relationship between treatment outcomes and recurrent lesions, multiplicity of risk factors, long-standing disease, and pathologic type. There was also a significant association between the number of surgical excisions and multiplicity of risk factors, as well as recurrence, location, and size of basal cell carcinoma.

**Study limitations:**

Lack of magnetic resonance imaging assessment in cases of suspected perineural invasion.

**Conclusions:**

High-risk basal cell carcinoma had a high cure rate by staged excision. Patients with more risk factors and those with nasal and recurrent basal cell carcinoma required more staged excisions. Failure of treatment is more probable in patients with more risk factors, long-standing lesions, and high-risk pathologic and recurrent basal cell carcinomas.

## Introduction

Basal cell carcinomas (BCCs) are the most common malignancy, with an increasing global incidence in recent decades. However, even though the mortality rate due to BCCs is very low, treatment-related costs impose an important burden on the health care system.^1^, ^2^

Although the majority of BCCs are easily cured, high-risk basal cell carcinomas (HRBCCs), which represent a significant proportion of BCCs, require special attention to be eradicated.^2^, ^3^

Mohs micrographic surgery (MMS) is the treatment of choice for HRBCCs, but this method requires special instruments and both surgical and pathologic experts; it is also expensive.^4^, ^5^, ^6^

Staged excision with pathologic margin control (SEPMC) is a simple and accessible procedure with a high cure rate that is suggested for the treatment of special types of BCC or for special sites such as periorbital or nasal areas.^6^, ^7^, ^8^, ^9^, ^10^

Considering the very high prevalence of BCCs, the relatively high number of HRBCCs, and the lack of access to MMS at most dermatosurgery centers worldwide, the present study evaluated the results of SEPMC in HRBCCs in the head region over seven years.

## Methods

### Study design and population

This clinical, interventional, follow-up study was performed on 122 patients over a period of eight years from 2008 to 2016 at the Hajdaie dermatology clinic of Kermanshah University of Medical Sciences, Iran. A biopsy was performed for patients clinically suspected of BCC. After histopathologic documentation of BCCs and their type, the patients with HRBCC criteria were chosen as candidates for this treatment modality.

All participants were informed about this procedure and were asked for their consent. Then, they were recruited in the study.

The exclusion criteria included lesions with a diameter larger than 5 cm, penetration of malignant cells into deep subcutaneous area or cartilage involvement, extension of tumor to special sites such as conjunctiva, staged surgery more than four times, pregnancy, comorbidities such as immunodeficiency, and genetic susceptibility to BCCs such as xeroderma pigmentosum and repair abnormalities.

Demographic data, site, clinical and pathologic characteristics of BCCs, type and number of risk factors, treatment outcome, and complications were recorded on the questionnaires used in this study.

### Criteria for HRBCCs

HRBCCs were considered to be lesions in high-risk locations, pathologic and clinical types, lesions larger than 2 cm, those with perineural invasion, those with indeterminate borders, recurrent lesions, and those in patients less than 30 years old.

### Procedural methods

BCC lesions were assessed under appropriate lighting and 2.5× loupe magnification; their margins were determined by a surgical marker. In the case of lesions with undefined margin, a disposable and sharp curette was used under local anesthesia to remove the fragile and abnormal tumoral tissue in order to determine the margin.

After determining the tumor border and local anesthetics, the BCCs were excised with a 4 to 6 mm surgical margin based on the number of risk factors for BCCs. The excised tumoral tissue was marked with suture or a different ink color in the superior and inferior directions.

The excised and marked sample was fixated in 10 % formaldehyde solution and submitted to a pathology laboratory. The pathology department was instructed to present the histopathological results as soon as possible.

Subsequent tissue excision was advanced 2 to 3 mm at each stage based on the histopathologic report if the resection margins were found to be histopathologically positive, or when the margin was too narrow in the involved areas. There were two-to-four day intervals between surgical stages. In order to prevent secondary infection, oral antibiotics were prescribed.

Finally, when all surgical margins were tumor-free, the repair approach was established based on the size, location, and other factors of the remaining defect.

The number of surgical stages and probable complications during the surgical process was recorded in the questionnaire.

### Pathologic assessment

The specimens were received in 10% formaldehyde solution and were oriented according to their labels. Then, thin representative pieces were taken from the labeled tissue margin to diagnose the tumoral tissue involvement. After preparation of slides, staining was done by hematoxylin-eosin method. If any of the margins were involved according to the labels, the location of the involvement site was given to the dermatosurgeon and subsequent excision was recommended until free margins were obtained. The margins status was reported as soon as possible, not more than 48 hours.

### Ethical considerations

This study was approved by the Ethics Committee of Kermanshah University of Medical Science and registered in the IRCT database (IRCT201412196403N5). Patient information was kept confidential.

### Statistical analysis

Data analysis was performed by SPSS (v. 16) using descriptive and inferential statistics.

As for the descriptive statistics, the central tendency and the dispersion along with tables were reported.

Regarding inferential statistics, the chi-squared test and multiple logistic regression analysis were applied. Multiple logistic regression models were created to examine the relationship between study variables and outcome and number of excision steps to provide the odds ratio (OR) with a 95% confidence interval (95% CI). The level of significance was set at 0.05.

## Results

A total of 122 patients, 47 (38.5%) females and 75 (61.5%) males, were recruited in this study. The age range of the participants was 38‒81 years, with a mean age of 57.66 ± 9.13 years (Table 1).

**Table 1:** Characteristics of the study population.

Percent	Frequency		Variables
38.5	47	Female	Sex
61.5	75	Male	
58.2	71	< 60	Age (years)
41.8	51	≥ 60	
74.6	91	≤ 20	Size (mm)
25.4	31	> 20	
53.3	65	≤ 48	Duration (months)
46.7	57	> 48	
56.6	69	Negative	Personal history of BCC
43.4	53	Positive	
25.4	31	Type II	Skin phenotype
61.5	75	Type III	
13.1	16	Type IV	
40.2	49	Nose	Site
15.6	19	Periorbital	
8.2	10	Forehead	
7.4	9	Cheek	
5.7	7	Post-auricular	
4.9	6	Ear	
4.9	6	Lateral of face	
3.3	4	Chin	
3.3	4	Periorificial	
3.3	4	Nasolabial fold	
3.3	4	Scalp	
60.7	74	Defined	Border
39.3	48	Undefined	
63.1	77	Yes	Previous treatment
36.9	45	No	
82.0	100	Nodular	Clinical type
8.2	10	Superficial	
9.8	12	Morpheaform	
55.7	68	Nodular	Pathologic type
17.2	21	Infiltrative	
9.8	12	Morpheaform	
9.0	11	Micronodular	
8.2	10	Superficial	
13.9	17	I	Number of risk factors
47.5	58	II	
25.4	31	III	
13.1	16	IV	
7.4	9	I	Number of excision stages
45.9	56	II	
30.3	37	III	
16.4	20	IV	
89.3	109	Cure	Outcome of treatment
10.7	13	Failure	

BCC, basal cell carcinoma.

The nasal area was the most common site of treatment 49 (40.2%), and the periorbital area 19 (15.6%) and forehead 10 (8.4%) were other commonly treated areas (Table 1).

Clinically, 100 (82.0%), 12 (9.8%), and 10 (8.2%) BCCs manifested as nodular, superficial, and morphoeic types, respectively (Table 1).

Pathologic assessment of lesions revealed 44 (36.1%) high-risk and 78 (73.9%) low-risk pathologic types (Table 1).

Seventy-seven (63.1%) patients presented with primary BBCs and 45 (36.9%) patients had recurrent BCCs as a risk factor, who had been previously subjected to inappropriate procedures (Table 1).

Moreover, one-, two-, three-, and four-stage surgical excisions were done in nine (7.4%), 56 (45.9%), 37 (30.3%), and 20 (16.4%) lesions, respectively (Table 1).

Out of 122 lesions, 109 lesions (89.3%) were cured and only 13 lesions (10.7%) showed recurrence during the follow-up period (Table 1).

Other demographic and clinical characteristics of the patients are presented in Table 1.

No complications, including infection in the surgical or para-surgical sites and bleeding, were seen during the surgical and follow-up periods.

To better quantify the relationship between the study variables and the number of surgical excisions and treatment outcomes, a more concise classification of some of the variables was performed (Table 2).

**Table 2:** Univariate logistic regression analysis based on treatment outcomes and number of excision steps.

Outcome		Univariate			Number of excision stages		Univariate
95% CI	OR	*p*-value	*≤*2	> 2	95% CI	OR	*p*-value
‒	1	0.545	30 (63.8)	17 (36.2)	‒	1	0.066
0.198‒2.35	0.682		35 (46.7)	40 (53.3)	0.954‒4.26	0.496	
‒	1	0.264	31 (47.7)	34 (52.3)	‒	1	0.188
0.602‒6.37	0.510		34 (59.6)	23 (40.4)	0.301‒1.26	1.62	
‒	1	0.106	21 (42.9)	28 (57.1)	‒	1	0.006
0.115‒1.23	2.56		44 (60.3)	29 (39.7)	0.237‒1.03	2.02	
‒	1	0.388	43 (47.3)	48 (52.7)	‒	‒	0.025
0.813‒8.65	1.99		22 (71.0)	9 (29.0)	1.13‒6.56	2.7	
‒	1	0.031	46 (70.8)	19 (29.2)	‒	‒	0.065
0.059‒0.873	0.227		19 (33.3)	38 (66.7)	0.096‒0.445	0.207	
‒	1	0.057	40 (58.0)	29 (42.0)	‒	‒	0.236
0.087‒1.03	0.301		25 (47.2)	28 (52.8)	0.315‒1.33	0.647	
‒	1	0.001	37 (54.4)	31 (45.6)	‒	‒	0.778
0.928‒11.03	0.313		28 (51.9)	26 (48.1)	0.667-1.03	1.1	
‒	1	0.052	9 (40.9)	13 (59.1)	‒	‒	0.203
0.086-1.01	3.3		56 (56.0)	44 (44.0)	0.720‒4.69	1.8	
‒	1	0.017	62 (80.5)	15 (19.5)	‒	‒	0.005
0.063‒0.760	0.219		3 (6.7)	42 (93.3)	0.005‒0.063	0.017	
‒	1	0.167	41 (55.4)	33 (44.6)	‒	‒	0.559
1.16‒13.9	0.248		24 (50.0)	24 (50.0)	0.389‒1.66	0.805	
‒	1	0.006	48 (63.2)	28 (36,8)	‒	‒	0.006
0.038‒0.571	0.148		17 (37.0)	29 (63.0)	0.160‒0.730	0.342	

OR, odd ratio; 95% CI, 95% confidence interval.

The results of logistic regression analysis showed a significant relationship between the number of surgical excisions and tumor location, multiplicity of risk factors, tumor size, and recurrent lesions (p < 0.05; Table 2).

The results of logistic regression also showed a significant relationship between treatment outcome and pathologic type, recurrent cases of BCCs, multiplicity of risk factors, and disease duration (p < 0.05; Table 2).

## Discussion

This study showed a 89.3% cure rate for HRBCCs through SEPMC, with a mean of 2.63 stages of excision over 48 months of follow-up.

Patients with a tumor size more than 20 mm, nasal BCCs, recurrent BCCs, and those with more than two risk factors required more surgical excisions.

The results of this study showed that failure of SEPMC was more possible in the high-risk pathologic types, recurrent BCCs, multiple risk factors, and long-standing diseases.

In the present study, patients with recurrent BCCs and more risk factors required more surgical excision and had more possibility of treatment failure. Therefore, patients with the mentioned variables require more attention for a complete cure.

Several studies have indicated 95% cure rate for BCCs through SEPMC.^6^, ^7^, ^8^, ^9^

The present study also showed a nearly 90% successful treatment rate for HRBCCs by SEPMC.

It is believed that this discrepancy is due to the presence and multiplicity of the risk factors, particularly recurrent BCCs. In addition, in some previous studies, SEPMC has been performed in either low- and high-risk BCCs or specific areas such as the nose or periorbital area.^7^, ^8^, ^9^

The factors that cause treatment failure through SEPMC include main tumors with skip areas, perineural invasion of some high-risk BCCs, and inconsistency between the surgeon and the pathologist in marking the tissue samples for pathologic assessment.^11^, ^12^

It appears that recurrent BCCs lead to irregular growth and expansion of tumor islands from the primary tumor location, making them difficult to cure by SEPMC and even by MMS.^10^ Many studies have reported the successful treatment of recurrent BCCs is less probable than that of the primary BCCs, even through MMS, and requires more surgical excisions, which is consistent with the current findings.^13^, ^14^, ^15^

Obviously, in cases of the disease with delayed treatment, the lesion is associated with an increased size, deeper penetration, and more possibility of skip areas and metastasis.^1^, ^16^, ^17^ Therefore, treatment failure is more likely in cases with a long evolution, and especially after ordinary surgical excisions.

In the present study, nasal lesions had a higher number of surgical excisions, which is consistent with the results of many previous studies. Nasal BCCs are more likely to develop fan-like dissemination and perineural invasion. There are also more pathologic and clinically high-risk cases that increase the number of surgical procedures.^5^, ^18^, ^19^

BCCs with high-risk pathologic features – including morphoeic, infiltrative, and micronodular types – are more likely to develop skip area dissemination and perineural invasion; therefore, recurrence is possible even with MMS.^2^, ^11^, ^12^, ^13^, ^16^, ^17^

In the current study, patients with high-risk pathologic lesions showed a significantly higher recurrence rate than those with low-risk pathologic lesions despite staged surgical excisions.

## Conclusion

Treatment of HRBCCs through SEPMC resulted in a high cure rate. Patients with more than two risk factors and recurrent lesions required a higher number of surgical excisions and had a higher possibility of treatment failure.

Patients with BCCs larger than 20 mm and nasal lesions required a greater number of surgical excisions. BCCs with high-risk pathologic type and duration of more than 48 months were associated with more possibility of treatment failure. The authors suggest new studies for better assessment of the findings of the present study.

## Financial support

None declared.

## Authors’ contributions

Reza Kavoussi: Drafting and editing of the manuscript; collection, analysis, and interpretation of data.

Hossein Kavoussi: Approval of final version of the manuscript; conception and planning of the study; participation in the study design.

Ali Ebrahimi: Participation in the study design; intellectual participation in the propaedeutic and/or therapeutic conduct of the studied cases; critical review of the literature; critical review of the manuscript.

Nader Salari: Statistical analysis; critical review of the manuscript.

Seyed Hamid Madani: Conception and planning of the study; collection, analysis, and interpretation of data.

## Conflicts of interest

None declared.

## References

[1] Ocanha J.P., Dias J.T., Miot H.A., Stolf H.O., Marques M.E., Abbade L.P. (2011). Relapses and recurrences of basal cell face carcinomas. An Bras Dermatol.

[2] Puig S., Berrocal A. (2015). Management of high-risk and advanced basal cell carcinoma. Clin Transl Oncol.

[3] Hamid O., Goldenberg G. (2013). Identifying patients at risk for recurrent or advanced BCC. J Drugs Dermatol.

[4] Cernea S.S., Gontijo G., Pimentel E.R., Tarlé R.G., Tassara G., Ferreira J.A. (2016). Indication guidelines for Mohs micrographic surgery in skin tumors. An Bras Dermatol.

[5] Kavoussi H., Ebrahimi A. (2013). Treatment and cosmetic outcome of superpulsed CO2 laser for basal cell carcinoma. Acta Dermatovenerol Alp Pannonica Adriat.

[6] Durmus Ucar A.N., Durmus Kocaaslan F.N., Salman A., Demirkesen C., Erdem Bayram F. (2019). Bayramicli M. margin-controlled, staged surgical excision in the treatment of high-risk basal cell carcinomas of the head and neck region. J Cutan Med Surg.

[7] McGrath L.A., Meeney A., Currie Z.I., Mudhar H.S., Tan J.H. (2019). Staged excision of primary periocular basal cell carcinoma: absence of residual tumour in re-excised specimens: a 10-year series. Br J Ophthalmol.

[8] Eskiizmir G., Gencoglan G., Temiz P., Hircin Z., Ermertcan A. (2011). Staged-surgery with permanent pathology for the management of high-risk nonmelanoma skin cancer of the nose. Eur Arch Otorhinolaryngol.

[9] Husler R., Schlittler F.L., Kreutziger J., Streit M., Banic A., Schoni-Affolter F. (2008). Staged surgical therapy of basal cell carcinoma of the head and neck region: an evaluation of 500 procedures. Swiss Med Wkly.

[10] Ebrahimi A., Rezaei M., Kavoussi R., Eidizadeh M., Madani S.H., Kavoussi H. (2014). Superpulsed CO_2_ laser with intraoperative pathologic assessment for treatment of periorbital basal cell carcinoma involving eyelash line. Dermatol Res Pract.

[11] França K., Alqubaisy Y., Hassanein A., Nouri K., Lotti T. (2018). Histopathologic pitfalls of Mohs micrographic surgery and a review of tumor histology. Wien Med Wochenschr.

[12] Takata Pontes L., Fantelli Stelini R., Cintra M.L., Magalhães R.F., Velho P.E., Moraes A.M. (2015). The importance of superficial basal cell carcinoma in a retrospective study of 139 patients who underwent Mohs micrographic surgery in a Brazilian university hospital. Clinics (Sao Paulo).

[13] Smeets N.W., Kuijpers D.I., Nelemans P., Ostertag J.U., Verhaegh M.E., Krekels G.A. (2004). Mohs’ micrographic surgery for treatment of basal cell carcinoma of the face ‒ results of a retrospective study and review of the literature. Br J Dermatol.

[14] Mosterd K., Krekels G.A., Nieman F.H., Ostertag J.U., Essers B.A., Dirksen C.D. (2008). Surgical excision *versus* Mohs’ micrographic surgery for primary and recurrent basal-cell carcinoma of the face: a prospective randomised controlled trial with 5-years’ follow-up. Lancet Oncol.

[15] Veronese F., Farinelli P., Zavattaro E., Zuccoli R., Bonvini D., Leigheb G. (2012). Basal cell carcinoma of the head region: therapeutical results of 350 lesions treated with Mohs micrographic surgery. J Eur Acad Dermatol Venereol.

[16] Asilian A., Tamizifar B. (2005). Aggressive and neglected basal cell carcinoma. Dermatol Surg..

[17] Ozgediz D., Smith E.B., Zheng J., Otero J., Tabatabai Z.L., Corvera C.U. (2008). Basal cell carcinoma does metastasize. Dermatol Online J.

[18] Wollina U., Bennewitz A., Langner D. (2014). Basal cell carcinoma of the outer nose: Overview on surgical techniques and analysis of 312 patients. J Cutan Aesthet Surg.

[19] Robinson J.K., Fisher S.G. (2000). Recurrent basal cell carcinoma after in-complete resection. Arch Dermatol.

